# Severe iodinated contrast extravasation following coronary CT angiography: A case report

**DOI:** 10.1016/j.radcr.2026.04.090

**Published:** 2026-05-27

**Authors:** Langhuan Yang, Chun'e Duan, Huimei Zeng, Shuyuan Xiong, Zixuan Zhou, Yan Luo, Li Dong

**Affiliations:** The First Affiliated Hospital of Yunnan University of Chinese Medicine/Yunnan Provincial Hospital of Traditional Chinese Medicine, Kunming 650310, Yunnan, China

**Keywords:** Coronary CT angiography, Iodinated contrast extravasation, Soft tissue injury, Subcutaneous effusion, Puncture and drainage, Case report

## Abstract

Severe iodinated contrast extravasation is a recognized complication of coronary computed tomography angiography (CCTA) that can progress to significant soft tissue injury if not promptly managed. We report the case of a 66-year-old female patient with a history of lung cancer surgery who developed severe contrast extravasation following the high-pressure injection of iohexol via a dorsal hand vein. The injury rapidly progressed to tension blisters and extensive subcutaneous effusion. Comprehensive staged management was implemented, beginning with acute cold compresses, escalating to timely minimally invasive puncture and drainage of 40 mL of liquefied necrotic tissue on day 8, and concluding with supportive topical wound care. After 27 days of integrated management, the swelling completely resolved with full restoration of skin integrity and no functional impairment. This case highlights that severe contrast extravasation may progress from inflammatory edema to tissue liquefaction, underscoring the necessity of early recognition and timely decompression to prevent necrosis and preserve hand function.

## Introduction

Coronary computed tomography angiography (CCTA) requires the rapid injection of iodine-based contrast medium at high flow rates (typically 4–5 mL/s), exerting significant pressure on the vascular walls. This can increase the risk of contrast medium extravasation, a known complication, particularly in patients with compromised vascular integrity. Cancer patients are especially vulnerable due to vascular sclerosis, often resulting from prolonged chemotherapy and nutritional depletion, which increase the fragility of blood vessels [1]. Extravasation of iodine-based contrast agents leads to local tissue swelling and pain, but if not properly managed, it can result in tension blisters, subcutaneous effusion, and, in severe cases, compartment syndrome and skin necrosis [2]. This report presents a case of severe contrast extravasation during CCTA via a dorsal hand vein complicated by extensive subcutaneous effusion, successfully managed through timely escalation of intervention and staged multidisciplinary care, highlighting the importance of early recognition and appropriate management to prevent serious soft tissue complications.

## Case presentation

### General information

A 66-year-old female patient presented to the outpatient clinic with a 3-month history of recurrent exertional chest tightness, typically lasting 5–10 minutes per episode and relieved by rest. She had a history of left lung adenocarcinoma and underwent a left lower lobectomy and mediastinal lymph node dissection 7 months prior. This was followed by two cycles of adjuvant chemotherapy, which, along with her post-operative status, significantly contributed to her fragile vascular condition and systemic cachexia. Given her intermediate risk profile and preference for non-invasive screening, CCTA was selected to evaluate suspected coronary artery disease.

Initial laboratory investigations revealed mild hyperlipidemia with a total cholesterol of 5.32 mmol/L (normal range: < 5.18 mmol/L) and low-density lipoprotein cholesterol (LDL-C) of 3.75 mmol/L (normal: < 3.37 mmol/L). Other notable findings included hyperuricemia (uric acid 430 μmol/L, normal: 150-360 μmol/L), mildly elevated fasting glucose (6.85 mmol/L, normal: 3.9-6.1 mmol/L), and slight transaminitis (ALT 47 U/L, normal: 7-40 U/L; GGT 51 U/L, normal: 7-45 U/L). Renal function, including serum creatinine, and coagulation profiles were within normal limits. Blood rheology tests indicated a hyperviscosity state. These findings, specifically the elevated ALT and GGT, were suggestive of mild hepatic steatosis (fatty liver), while the fasting glucose level indicated a prediabetic state, for which the patient was advised to undergo further endocrinology screening.

### Vascular assessment and catheterization

Nursing assessment revealed extremely poor systemic vascular conditions, characterized by thin and inelastic vessels. No suitable straight vessels of adequate caliber, such as the median cubital vein, could be located. Consequently, a superficial venous catheterization was performed on the dorsum of the right hand using a 20G standard Y-type indwelling needle. Patency was confirmed with a saline flush, and the catheter was secured.

### Event description

On January 9, 2025, at 14:20, the patient underwent CCTA in the CT suite. A test injection of 20 mL normal saline was administered at 4 mL/s without incident. Subsequently, 40 mL of the non-ionic iodinated contrast agent iohexol (350 mgI/mL) was injected via a high-pressure injector using a dual-syringe system. A saline chase was performed as per protocol. During the injection, a significant delay in contrast opacification at the aortic monitoring level, 2 cm below the carina, was observed, indicating inadequate intravascular delivery of the contrast agent, which raised immediate suspicion of contrast extravasation. Injection was immediately halted, and upon examination, swelling was observed at the base of the fingers on the dorsum of the patient's right hand. The procedure was terminated, and physical examination revealed obvious swelling with high tension and associated pain. The examination was deemed unsuccessful due to insufficient delivery of the effective contrast dose.

## Clinical management and interventions

The clinical course and management in this case spanned 27 days. Due to progressive swelling, blister formation, subcutaneous effusion, and delayed soft tissue recovery, the patient was managed with a staged approach including early diffusion control, timely decompression when liquefaction was suspected, infection prevention, and supportive wound care. Pain severity was assessed using the Numeric Rating Scale (NRS) throughout the course to monitor symptom progression and response to interventions.

### Acute phase: containment and emergency management

Upon detection of suspected contrast extravasation during CCTA on January 9, the power injection was immediately stopped. Given the visually explicit clinical signs of high-tension swelling and acute pain immediately following the extravasation, an immediate plain radiograph was not performed to spare the patient from additional and unnecessary radiation exposure for a clinically obvious diagnosis. The patient reported burning pain on the dorsum of the right hand (NRS 7). The clinical team aspirated approximately 2 mL of mixed contrast medium and blood through the retained catheter before removal. Excessive manual pressure was avoided to reduce further dispersion. Cold compresses with 50% magnesium sulfate solution were then applied continuously for 24 hours to limit diffusion and reduce local swelling.

### Inflammatory exudation phase and remote guidance (day 1-day 7)

From January 10 to 14, tense blisters developed on the dorsum of the hand with localized erythema and swelling. The patient remained at home during this period, and telephone follow-up indicated persistent distending pain (NRS 5–6). Due to limited ability to attend daily outpatient visits, remote follow-up was performed and supportive topical wound care was initiated. On day 5, recurrent blistering with spontaneous rupture suggested ongoing deep exudation. The patient was strongly advised to return for in-person reassessment.

### Necrosis and liquefaction phase: puncture drainage and anti-infection (day 8-day 13)

The patient returned to the hospital on January 17 (day 8). Physical examination revealed that while superficial swelling had subsided, significant fluctuation was palpable in the deep tissues, accompanied by severe pain upon pressure. The NRS score peaked at 8, indicating liquefaction of necrotic subcutaneous tissue and formation of an effusion.

#### Puncture and drainage

Following strict disinfection, multiple punctures were performed at sites of maximal fluctuation using a 5 mL sterile syringe. Approximately 30 mL of dark red, viscous bloody fluid and liquefied necrotic tissue were drained. The patient reported immediate relief of distending pain post-drainage, with the NRS score decreasing to 4.

#### Combined anti-inflammatory therapy

After debridement of superficial eschar, Bactroban Ointment (Mupirocin) was applied and covered with a silver-ion dressing to leverage the potent bactericidal and absorptive properties of silver ions for controlling deep-seated infection. Oral administration of Cefaclor (Cector) was also prescribed for 3 days as per medical orders.

#### Secondary drainage

On January 20 (day 11), re-examination showed significantly reduced fluctuation. A second puncture yielded approximately 10 mL of dark red fluid. As exudation diminished, the silver-ion dressing was discontinued.

### Granulation and repair phase: in-depth application of Zilian Ointment (Day 14-Day 27)

**Fig. 1:**
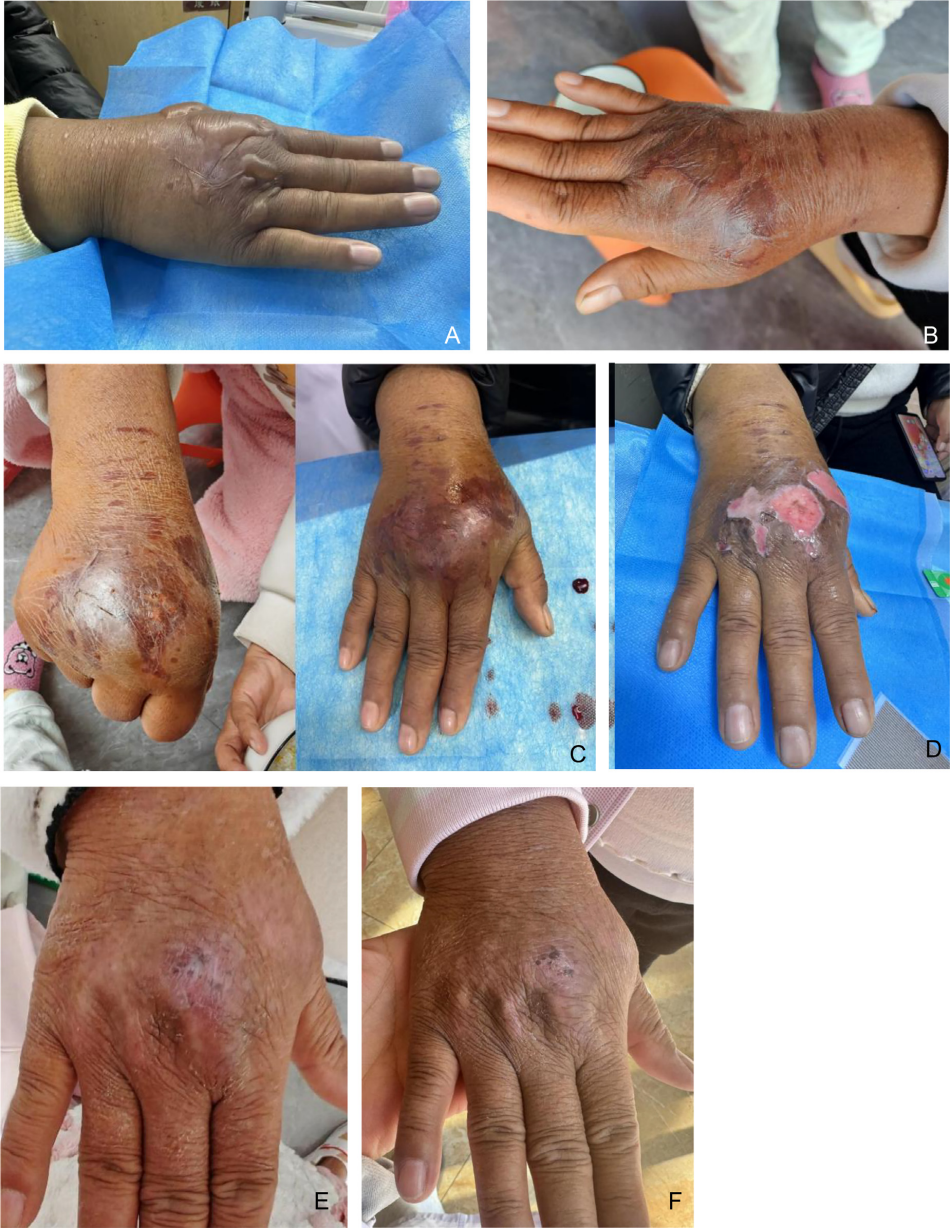
(A) On day 1 after the extravasation, blisters appeared on the patient's skin. (B) On day 5 after the extravasation, blisters reappeared on the patient's skin. (C) On day 7, follow-up assessment suggested a small amount of purulent discharge, and the patient was advised to seek prompt medical attention. (D) On day 8 after the extravasation, the patient returned to the hospital for a follow-up examination. Puncture drainage was performed, and the eschar was debrided. (E) On day 23 after the extravasation, a telephone follow-up revealed that the patient reported improvement in the skin condition with the regular application of Zilian Ointment. (F) On day 27 after the extravasation, the patient's skin surface was intact without ulceration, with scarring awaiting further resolution.

Beginning on day 14, the wound entered the repair phase. Pain decreased to mild intensity (NRS 2–3), with residual finger numbness and mild discomfort during movement. Supportive topical wound care with Zilian Ointment was continued during this phase to promote granulation and epithelialization. Progressive improvement in swelling, skin texture, and hand function was observed. By day 27, skin integrity on the dorsum of the hand was fully restored, subcutaneous induration had resolved, and hand function returned to baseline. No ulceration or further necrosis occurred (Fig. 1).

### Radiological follow-up

**Fig. 2:**
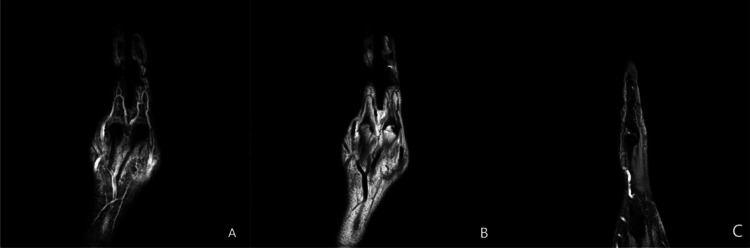
Follow-up magnetic resonance imaging (MRI) of the right hand on day 20 post-extravasation. (A) Coronal fluid-sensitive sequence (eg, T2-weighted/STIR) demonstrating resolving mild hyperintense signal (edema) primarily within the subcutaneous tissues of the dorsum of the hand. (B) Coronal T1-weighted sequence showing intact deep fascial planes and tendon structures without evidence of deep tissue necrosis or fluid collection. (C) Sagittal sequence confirming that inflammatory changes remain confined to the superficial soft tissues, with no signs of osteomyelitis or deep compartmental involvement.

To objectively evaluate the extent of soft tissue recovery and rule out deep space infections, a follow-up magnetic resonance imaging (MRI) of the right hand was performed free of charge on day 20 post-extravasation (Fig. 2). The coronal and sagittal MRI sequences demonstrated resolving subcutaneous edema and residual inflammatory changes, primarily confined to the superficial soft tissues. Crucially, the deep fascial planes remained intact, and there was no radiological evidence of deep tissue necrosis, deep abscess formation, or osteomyelitis. These imaging findings correlated well with the patient's positive clinical progression and tissue repair.

## Discussion

The purpose of this case report is to highlight that severe contrast extravasation during CCTA can progress insidiously from inflammatory edema to tissue liquefaction. We aim to propose a staged management protocol—integrating early recognition, minimally invasive decompression, and supportive wound care—to prevent irreversible tissue necrosis.

Contrast extravasation is a recognized complication of coronary computed tomography angiography (CCTA), particularly due to the requirement for high-flow, high-pressure contrast injection. In this case, the patient's markedly compromised venous condition following lung cancer surgery necessitated the use of a dorsal hand vein, which is anatomically characterized by thin vessel walls, limited muscular support, and loose surrounding connective tissue. These features render it particularly vulnerable to sudden hemodynamic stress generated by power injectors [3]. Since adequate coronary artery opacification strictly requires high flow rates (typically 4-5 mL/s), utilizing a dorsal hand vein in patients with exhausted vascular access becomes a calculated clinical risk driven by diagnostic necessity.

However, taking such a risk demands tailored technical execution, and it must be acknowledged that our injection protocol presented a significant oversight. While driven by the technical requirement for optimal coronary opacification, maintaining the standard high flow rate (4 mL/s) via a fragile dorsal hand vein exacerbated the patient's risk. Ideally, for distal sites like the dorsal hand, the flow rate should be limited to ≤ 2 mL/s, or the procedure should be delayed until a more proximal large vein can be accessed. The failure to down-titrate the flow rate in this instance serves as a critical reminder for imaging departments: when standard venous access is unavailable and clinical necessity forces a compromise, site-specific flow rate adjustments and extreme continuous visual monitoring are absolutely critical.

The reported overall incidence of contrast media extravasation during contrast-enhanced CT scans ranges from 0.1% to 0.9% [4]. While most instances result in mild, self-limiting localized pain and erythema, severe complications such as skin necrosis, ulceration, or acute compartment syndrome are rare but potentially devastating. The severity of the injury is partly dose-dependent; studies indicate that large volumes of extravasated contrast (typically > 50 mL) are significantly associated with a higher risk of severe tissue damage. In the present case, the extravasation of approximately 40 mL of hyperosmolar iohexol into the confined and delicate subcutaneous space of the dorsal hand initiated a rapid inflammatory cascade. This underscores that in patients with difficult venous access—such as those with a history of long-term chemotherapy, advanced age, or cachexia—more stringent venous assessment and site selection are required prior to high-flow imaging examinations such as CCTA.

A critical turning point in this case occurred on day 8, when a distinct fluctuant sensation accompanied by severe pain (NRS score 8) was detected, and aspiration yielded 30 mL of dark, old effusion. This clinical presentation suggested progression from inflammatory edema to tissue necrosis and liquefaction. The hyperosmolar nature of iohexol plays a central role in this process, promoting continuous fluid shifts into the interstitial space, resulting in cellular dehydration, apoptosis, and the formation of a high-tension fluid cavity. At this stage, continued conservative management alone may exacerbate microcirculatory compromise, increasing the risk of compartment syndrome or skin necrosis [5].

Our staged management approach aligns with established clinical protocols. The American College of Radiology (ACR) Manual on Contrast Media recommends conservative initial management, including limb elevation and the application of cold compresses, to decrease local inflammatory responses and limit the diffusion of the vesicant [6]. However, when continuous monitoring reveals failure of conservative management—evidenced by recurrent blistering, high tissue tension, and palpable fluctuance—prompt intervention is mandatory. Literature indicates that timely surgical decompression or drainage is essential in these scenarios to prevent irreversible tissue necrosis or acute compartment syndrome [7]. Timely minimally invasive puncture and drainage therefore functioned as a form of “surgical decompression,” effectively relieving tissue pressure, restoring capillary perfusion, and creating favorable conditions for subsequent tissue repair.

In addition to decompression and infection control using silver ion dressings, adjunctive topical therapy with Zilian Ointment was introduced during the subacute and recovery phases to support wound healing. Rather than replacing standard management, this intervention served as a complementary strategy aimed at controlling residual inflammation and facilitating granulation and epithelialization. During the early phase, its application was associated with alleviation of erythema, swelling, and pain related to chemical phlebitis, while in the later phase it appeared to support progressive tissue repair and scar softening. Although the precise mechanisms require further investigation, this staged topical approach may offer a practical option for managing delayed healing following severe contrast extravasation [8,9].

In summary, this case highlights that severe iodinated contrast extravasation following CCTA requires timely escalation beyond conservative measures when clinical signs suggest tissue liquefaction and rising compartmental pressure. An integrated management strategy consisting of early diffusion control, mid-term decompression through puncture and drainage, and supportive wound care may effectively prevent necrosis and long-term functional impairment. This experience provides a valuable reference for the imaging-related risk management and multidisciplinary care of high-risk patients undergoing CCTA.

## Ethical approval

This case report was conducted in accordance with the ethical standards of the institutional research committee and the 1964 Helsinki Declaration and its later amendments. Due to the retrospective nature of this case report involving a single patient and the use of anonymized clinical data, formal approval from the Institutional Ethics Committee was not required by our hospital policy.

## Patient consent

Written informed consent was obtained from the patient for the publication of this case report, including all related clinical images and medical details.

## References

[1] Nagayama Y., Nakaura T., Awai K. (2025). Contrast medium dose optimization in the era of multi-energy CT[J]. Japanese J Radiol.

[2] Talasila P., Hedge S.G., Periasamy K. (2024). Imaging in esophageal cancer: a comprehensive review. Indian J Radiol Imaging.

[3] Fischer A.M., Riffel P., Henzler T. (2020). More holes, more contrast? Comparing an 18-gauge non-fenestrated catheter with a 22-gauge fenestrated catheter for cardiac CT. PloS One.

[4] Kodzwa R. (2019). ACR Manual on contrast media: 2018 updates. Radiol Technol.

[5] Liu Q., Huang F., Xiong X. (2025). Correlation between the degree of extravasation of non-ionic iodinated contrast media and clinical baseline characteristics of patients undergoing contrast-enhanced CT scans [J]. Chinese General Practice Nurs.

[6] Sbitany H., Koltz P.F., Mays C. (2010). CT contrast extravasation in the upper extremity: strategies for management. Int J Surg.

[7] Jia Y.F., Cui M.M., Li D. (2024). Analysis of influencing factors for contrast media extravasation during contrast-enhanced CT examinations based on logistic regression [J]. J Pract Med Imaging.

[8] Liu Y. (2025). Clinical study on Kangfuxin liquid combined with concentrated growth factor in the treatment of chronic skin ulcers [D]. HUCM.

[9] Li Y.C., Sun Y.M., Wang Y.W. (2023). Mechanistic studies on the therapeutic effects of Zilian Ointment on psoriasis in rats [J]. J Yunnan Minzu Uni (Nat Sci Ed).

